# Diversification of the Light-Harvesting Complex Gene Family via Intra- and Intergenic Duplications in the Coral Symbiotic Alga *Symbiodinium*

**DOI:** 10.1371/journal.pone.0119406

**Published:** 2015-03-05

**Authors:** Shinichiro Maruyama, Eiichi Shoguchi, Nori Satoh, Jun Minagawa

**Affiliations:** 1 Division of Environmental Photobiology, National Institute for Basic Biology, Okazaki, Aichi, Japan; 2 Marine Genomics Unit, Okinawa Institute of Science and Technology Graduate University, Onna, Okinawa, Japan; 3 Department of Basic Biology, School of Life Science, The Graduate University for Advanced Studies, Okazaki, Aichi, Japan; 4 Core Research for Evolutional Science and Technology (CREST), Japan Science and Technology Agency (JST), Kawaguchi, Saitama, Japan; University of Hyderabad, INDIA

## Abstract

The light-harvesting complex (LHC) is an essential component in light energy capture and transduction to facilitate downstream photosynthetic reactions in plant and algal chloroplasts. The unicellular dinoflagellate alga *Symbiodinium* is an endosymbiont of cnidarian animals, including corals and sea anemones, and provides carbohydrates generated through photosynthesis to host animals. Although *Symbiodinium* possesses a unique LHC gene family, called chlorophyll *a*-chlorophyll *c_2_*-peridinin protein complex (acpPC), its genome-level diversity and evolutionary trajectories have not been investigated. Here, we describe a phylogenetic analysis revealing that many of the LHCs are encoded by highly duplicated genes with multi-subunit polyprotein structures in the nuclear genome of *Symbiodinium minutum*. This analysis provides an extended list of the LHC gene family in a single organism, including 80 loci encoding polyproteins composed of 145 LHC subunits recovered in the phylogenetic tree. In *S*. *minutum*, 5 phylogenetic groups of the *Lhcf*-type gene family, which is exclusively conserved in algae harboring secondary plastids of red algal origin, were identified. Moreover, 5 groups of the *Lhcr*-type gene family, of which members are known to be associated with PSI in red algal plastids and secondary plastids of red algal origin, were identified. Notably, members classified within a phylogenetic group of the *Lhcf*-type (group F1) are highly duplicated, which may explain the presence of an unusually large number of LHC genes in this species. Some gene units were homologous to other units within single loci of the polyprotein genes, whereas intergenic homologies between separate loci were conspicuous in other cases, implying that gene unit ‘shuffling’ by gene conversion and/or genome rearrangement might have been a driving force for diversification. These results suggest that vigorous intra- and intergenic gene duplication events have resulted in the genomic framework of photosynthesis in coral symbiont dinoflagellate algae.

## Introduction

Light harvesting complex (LHC) proteins are peripheral components of photosystem (PS) I and PSII and essential for receiving and transferring light energy to the core machinery of the photosystems as well as dissipating such energy as heat under excess light conditions [1,2]. LHC proteins typically possess three transmembrane helices and bind photosynthetic pigments such as chlorophylls and xanthophylls, and a variety of LHC protein families have evolved in photosynthetic eukaryotes. Genes encoding LHCs are phylogenetically conserved across eukaryotes and are conceivably derived from a cyanobacterial gene encoding the single transmembrane helix polypeptide Hlip [3]. Although the function and composition of the photosystems, including their LHCs, have been extensively studied using the model land plant *Arabidopsis thaliana* and the model green alga *Chlamydomonas reinhardtii*, far fewer studies have investigated LHCs in other lineages, e.g., chlorophyll *a*-binding LHCs encoded by *Lhcr* genes in ‘red lineage’ organisms including red algae and algae harboring secondary plastids of red algal origin, fucoxanthin chlorophyll *a*/*c*-binding LHCs (FCP) encoded by *Lhcf* genes in stramenopiles (e.g., diatoms, brown algae), and chlorophyll *a*-chlorophyll *c*_2_-peridinin protein complex (acpPC, also known as ACP or iPCP) in dinoflagellates [3,4]. Although chlorophyll-binding sites on three transmembrane helices are shared by almost all LHCs, canonical carotenoid-binding sites found in green plant LHCs are less or minimally conserved in the ‘red lineage’ LHCs [3,5,6].

Dinoflagellates are known for their unique cellular and genetic features. Their chloroplasts (plastids), which originated from ancestral red algal endosymbionts, are surrounded by three membranes, unlike the four-membrane-bound chloroplasts in other algae possessing secondary plastids of red algal origin, such as stramenopiles, cryptophytes and haptophytes [7]. Dinoflagellate LHCs are expressed as multi-unit polyproteins and cleaved into single protein units for functionalization [8]. Among eukaryotes, these unique characteristics are shared with few other eukaryotic taxa, e.g., euglenophytes. These distantly related algal lineages include secondary algae of green algal origin, which are believed to represent conspicuous examples of convergent evolution in different lineages [9]. The structural and functional roles of repetitive DNA have been extensively studied in many organisms [10], and duplicated genes are often considered a functional backup to compensate for the loss of the gene copies [11]. Dinoflagellates represent a ‘showcase’ for the molecular evolution of repetitive DNA and gene duplication, exemplified by their high gene copy numbers associated with unusual trans-splicing-mediated transcription from histone-lacking chromosomes [12–14]. One of the prominent characteristics unique to dinoflagellates is their possession of a class of water-soluble pigment-binding antenna protein, called peridinin chlorophyll *a* protein (PCP), showing no sequence similarity to any LHC-related proteins [15]. Genes encoding PCP are highly duplicated and arranged in tandem on a single chromosomal locus and are present in 5000 identical gene copies without introns in the dinoflagellate *Gonyaulax polyedra* [16]. The in vivo function and structural composition of LHCs and PCP as well as their mutual interaction in the dinoflagellate chloroplasts remain under debate [17,18].

A previous study of the dinoflagellate genus *Symbiodinium*, known to be a symbiont in cnidarian animals such as corals and sea anemones, showed that the expression of LHCs was decreased under heat stress, causing the loss of the light-harvesting antenna and the bleaching of the algal cells in a high temperature-sensitive *Symbiodinium* strain [19]. Nevertheless, much remains to be investigated regarding the antenna proteins of this genus at the genomic level, and it is unclear which LHC subfamily binds to which photosynthetic pigments in what ratio and in which photosystem complexes it is assembled. Recently, the nuclear and chloroplast minicircle genomes of *Symbiodinium minutum* were sequenced, illustrating its unique gene repertoire and genome structure [20,21]. This study was the first nuclear genome reported in photosynthetic alveolates, and the sequencing identified many duplicated nuclear-encoded ‘plastid-transferred’ genes [21], which were originally encoded in the plastid genome of a red algal endosymbiont in the ancestral dinoflagellate and then transferred to the nuclear genome via endosymbiotic gene transfer [22]. To our surprise, in addition to the duplicated plastid-related genes, over 100 gene models encoding LHCs were found in the nuclear genome of *S*. *minutum* [20,21].

In the green plant lineage, the land plant *Arabidopsis thaliana* possesses 5, 4 and 1 genes encoding the type I, II and III major trimeric LHCII polypeptides, respectively. These genes include duplicated gene family members, and each of 3 minor monomeric LHCIIs is encoded by a single gene in addition to 4 genes encoding LHCI polypeptides [23,24]. In the green alga *Chlamydomonas reinhardtii*, four major trimeric LHCII (type I-IV) polypeptides are encoded by 5, 1, 2 and 1 genes, respectively, and a single gene for each of the two minor LHCII polypeptides and 9 genes for LHCI are present [24,25]. In *Euglena gracilis*, the plastid of which is derived from a green algal endosymbiont acquired via secondary endosymbiosis, 11 LHCI and 10 LHCII protein-coding genes were identified through the expressed sequence tag survey [26]. Although the gene family repertoire in the red lineages is distinct from that of the green lineage [27], the number of genes are comparable: approximately 30 LHC homologs were found in the nuclear genome of the diatom *Thalassiosira pseudonana* [28] and, in an extreme case, only 3 LHC genes are present in the unicellular red alga *Cyanidioschyzon merolae* [29]. These findings highlight the exceptional abundance of the number of genes in *S*. *minutum* and lead to several questions: How have such a large number of genes evolved? How many subfamilies can these genes be classified into and have contributed to diversification in the evolutionary history of the LHC gene family? What can we infer about the historical pattern of the genome evolution in *Symbiodinium*? To answer these questions, comprehensive cataloging and classification at the genomic level is essential but has not been presented to date.

In this study, we conducted LHC-related gene mining analyses using the transcriptome and genome sequence data of the dinoflagellate *Symbiodinium*, which is not only of particular ecological and environmental importance but an emerging model dinoflagellate for studying the evolutionary trajectory of the unique photosynthetic eukaryotes and the relationships between animal and plant symbiosis [30]. Here, we present a genome-wide gene mining and cataloging to illustrate the diversity of the LHC gene family in *Symbiodinium* and discuss possible mechanisms that may have given rise to the highly duplicated gene family in complex eukaryotic genomes.

## Materials and Methods

### Sequence analysis and phylogenetic tree construction

Polypeptide sequences of the LHC proteins were collected from the genome sequence data of the coral symbiont dinoflagellate *Symbiodinium minutum* strain Mf1.05b.01 (Clade B1) (http://marinegenomics.oist.jp/genomes/gallery) [20] using the jackhammer program in the HMMER package (ver. 3.1b, http://hmmer.org/) and sym17_1, the amino-terminal half of an LHC protein in *Symbiodinium* sp. (Clade C3) (accession number CBI83422), as a query [5,31]. These sequences were then combined with the previously reported LHC proteins in *Symbiodinium* sp. C3 [5], the model diatoms *Phaeodactylum tricornutum* strain CCAP 1055/1 and *Thalassiosira pseudonana* strain CCMP1335 LHCs [32], and *Chlamydomonas reinhardtii* [24] as references. Multiple sequence alignment constructions and phylogenetic analyses were run as previously described [33]. Briefly, single-unit LHC genes were extracted and aligned using MAFFT [34] and TrimAl [35], and then maximum-likelihood (ML) trees were constructed using RAxML with 400 bootstrap resamplings [36]. The approximately ML tree was constructed, and its local support values with the Shimodaira-Hasegawa test were calculated using FastTree [37]. The unit structures of LHC genes were analyzed based on the RAxML and FastTree tree topologies.

### RNAseq read mapping onto gene models

The LHC domains predicted by HMMER and other conserved proteins were used to extract the corresponding coding DNA sequences (CDS) [5,20]. RNAseq read data for heat stress-treated and control cells [20] (DDBJ Sequence Read Archive [http://trace.ddbj.nig.ac.jp/dra/] accessions DRR003865-DRR003871) were mapped onto the CDS fragments using Bowtie 2 [38]. Heat maps of the reads per kilobase of transcript per million mapped reads (RPKM) onto each LHC protein unit were generated using the R package (http://www.r-project.org).

## Results and Discussion

**Fig 1:**
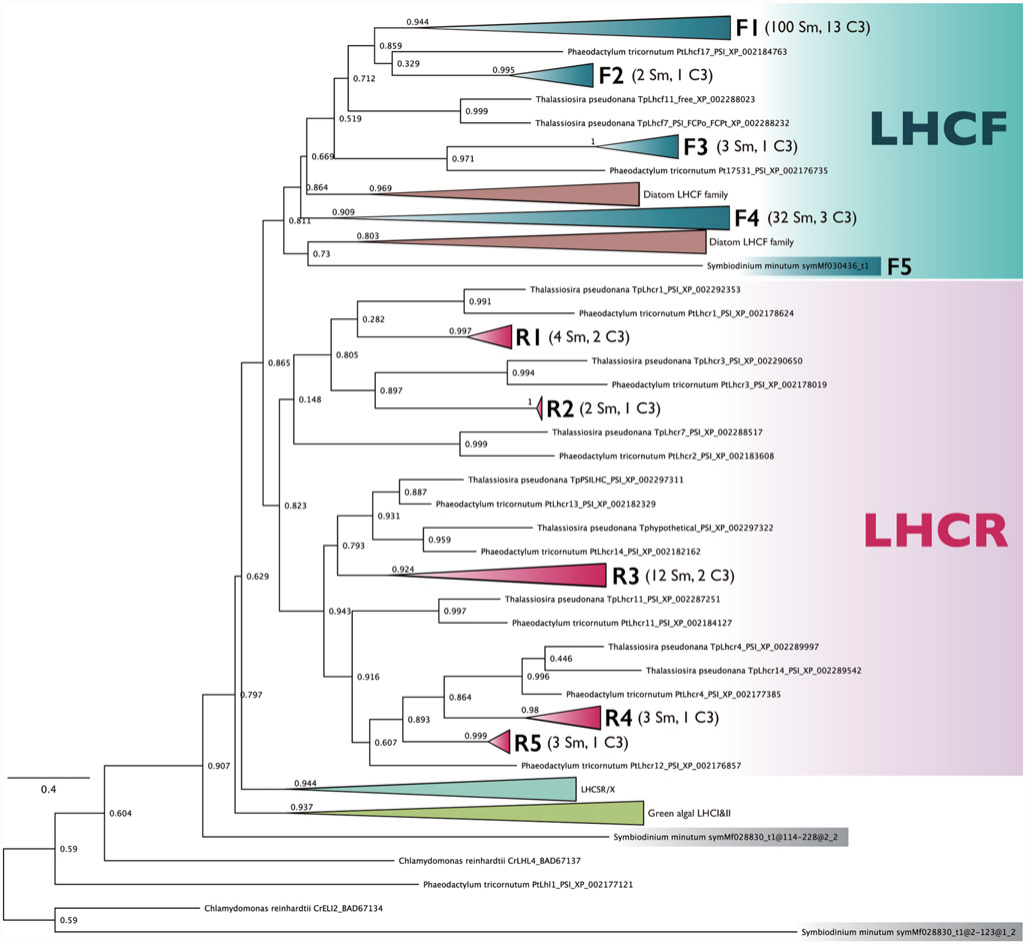
Phylogenetic tree of the *S*. *minutum* LHC proteins. Categorization of the LHC gene families is based on the clades supported by significant SH-like scores (0.9 or higher). Sm and C3 indicate the number of LHC proteins identified in *Symbiodinium minutum* and *Symbiodinium* sp. C3, respectively. See full tree in S1 Fig.

In the nuclear genome of *S*. *minutum* [20,21], many of the LHCs were encoded in highly duplicated nuclear genes with multi-unit structures. Although assembling highly duplicated genomic regions is a major challenge in genomics, paired-end sequencing of bacterial artificial chromosomes and fosmid libraries enabled us to assess the quality of assemblies of the *S*. *minutum* genome [20]. By using one of the LHC proteins in *Symbiodinium* sp. (Clade C3), sym17_1, as a query [5], we detected 199 LHC protein units from 92 loci with the jackhammer program. For phylogenetic analysis, we removed redundant polypeptide sequences derived from alternatively spliced RNAseq contigs and generated a dataset composed of 164 LHC proteins, with each encompassing three trans-membrane helices, out of 82 loci of genes encoding polyproteins. After multiple alignment and gap trimming, the resulting matrix included 145 non-redundant polypeptide sequences from 80 loci. Phylogenetic analysis showed that *S*. *minutum* possessed genes encoding three groups of LHC family proteins: LHCR-type, LHCF-type and a group composed of two miscellaneous LHC-like proteins encoded by a single gene locus (ID 028830) (Fig. 1, S1 Fig). Basal topologies were not fully resolved in the ML tree constructed by RAxML [36], likely due to the small sizes of LHC protein unit (118 amino acid length in the alignment used in this study). However, FastTree, originally designed to infer phylogenies for large alignments [37], assigned relatively high branch support values to major clades (Fig. 1), which is consistent with previous studies [7]. Thus, we used the clusters supported by FastTree for further discussion.

Our data showed the extensive diversifications of the two LHC gene subfamilies, *Lhcr*-type and *Lhcf*-type, each of which could be classified into 5 phylogenetic groups (phylogroups F1–5 and R1–5), whereas no homologs of stress-responsive *Lhcsr/Lhcx*-type genes and *PsbS*-type genes were identified in *S*. *minutum* (Fig. 1) [39,40]. In reference to the LHC clades in *Symbiodinium* sp. C3 proposed by Boldt et al. [5], the groups F1 and F2 presented here correspond to a phylogenetic clade recognized as ‘Clade 3b’; the groups F3 and F4 are equivalent to ‘Clade 3a’ and ‘2,’ respectively; and the groups R1–5 are ‘Clade 1.’ *Lhcr*-type is a chlorophyll *a*-binding LHC protein gene subfamily conserved among red algae and photosynthetic eukaryotes harboring secondary plastids of red algal origin. This subfamily includes *fcp4* genes in diatoms and corresponds to the clade III defined by Hoffman et al. [7]. *Lhcr* gene products have been shown to be associated with PSI in red algae [29,41,42] and diatoms [32,43], suggesting that the LHCR proteins conceivably play a major role in harvesting light for PSI in these organisms. Furthermore, *Lhcf*-type genes include members of FCPs, which were predominantly detected from free trimeric FCP complexes or higher oligomers detached from photosystem reaction centers in the pennate diatom *Phaeodactylum* [32,44], and were detected in both a trimeric FCP complex and PSII-FCP supercomplex in the centric diatom *Cyclotella* [43]. Considering the presence of peridinin instead of fucoxanthin in most dinoflagellates, including *Symbiodinium*, peridinin is most likely a major light-harvesting carotenoid pigment in LHCF-type LHC proteins in *Symbiodinium*. A recent study showed that peridinin was associated with the LHC protein complex fraction and that the pigment composition of chlorophyll *a*:chlorophyll *c*_2_:peridinin:diadinoxanthin was determined to be in the molecular ratio 4:6:6:1 in *Symbiodinium* sp. [6]. However, the types of LHC proteins that participate in each complex formation in the *Symbiodinium* photosystem have yet to be elucidated. Our results provide a roadmap for investigating how many and which LHC family members are involved in harvesting light in the photosystem, and identifying key players in photosynthesis from the entire catalog of the highly duplicated gene family.

**Fig 2:**
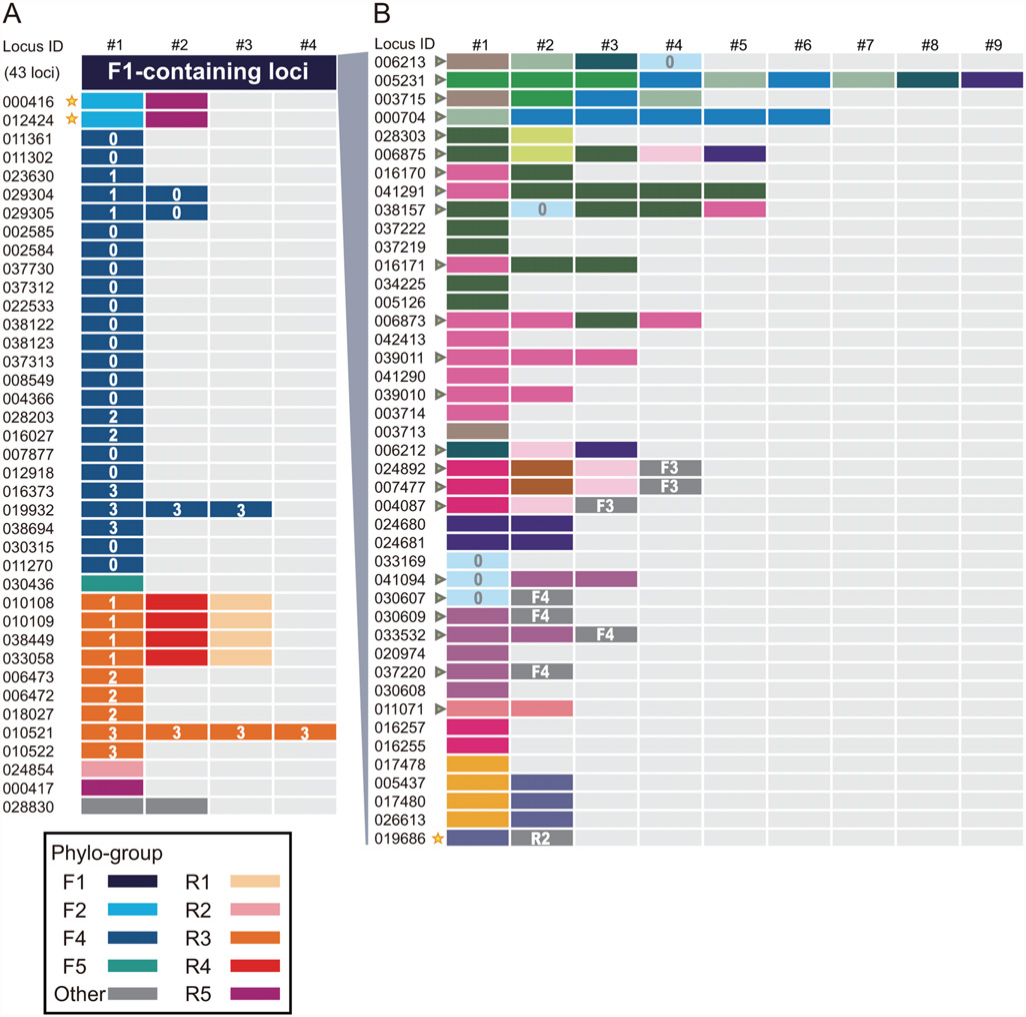
Composition and gene distribution of the *S*. *minutum* LHCR and LHCF proteins. (A) Different colors represent phylogenetic groups and the subgroupings within each group recovered in the phylogenetic tree in Fig 1. Group F1 is represented as a single box for clarity (see Fig. 2B). Numbers in the boxes indicate subgroupings within the F4 and R3 groups supported in the phylogenetic tree, where ‘0’ represents the F4 group members with no subgroupings found in the tree. (B) Detailed distribution pattern of group F1. Different color codes indicate subgroupings in the tree, with subgroup ‘0’ showing no phylogenetic affiliation with any other subgroups. Non-F1 members are shown in the gray boxes with the names of the groups. The arrowhead indicates the gene locus possessing the SPLR motif (see text). The star indicates the fusion gene of LHCR and LHCF families. Numbers with ‘#’ above the boxes indicate the unit labels for each protein unit in polyproteins.

Although still controversial, paralogs are defined as homologous genes that have evolved via gene duplication, whereas orthology describes the relationship between homologous genes that emerged via speciation [45]. To varying degrees, paralogs retain sequence homology with other members of the paralogous gene family, and the relationships can be interpreted from phylogenetic analysis. In this study, we recognized two modes of possible gene duplications for paralogous LHC gene units: intragenic duplication, in which the closest homolog of a gene unit was found nearby, namely, within the same locus, and intergenic duplication, in which the similarity between gene units located in physically separate loci was highest. Interestingly, based on our classification, only the group F1 is exceptionally highly duplicated; the duplication in the rest of the phylogenetic groups is comparable to that in other algal species, e.g., diatoms (Fig. 1). Thus, a large number of LHC gene loci in the *S*. *minutum* genome can be accounted for by conspicuous expansion of the phylogenetic group F1, which can be interpreted as the consequence of multiple rounds of intergenic and intragenic duplications within the group (Figs. 1 and 2). Recently, LHC antenna proteins were isolated from *Symbiodinium* sp. strain CS-156 (Clade C), and mass spectrometry analysis showed that the isolated protein sample had the most hits for the cDNA sequence (GenBank: FN646416.2) encoding a *Symbiodinium* sp. C3 LHC protein [46], which belongs to group F1 in our tree (Fig. 1). It is tempting to speculate that the highly duplicated group F1 may include a major antenna protein component or be present in a large quantity, to a certain degree proportional to the number of genes, compared to other groups. If so, why is this phylogenetic group, and LHCs in *S*. *minutum* as a whole, so highly duplicated? Although the lack of transgenic tools in dinoflagellates makes this question difficult to address, previous studies have suggested an apparent correlation between copy number and expression level [12–14]. This possibility allows us to consider that gene duplication may contribute to an elevated transcription level in a gene dosage-dependent manner and give the genes a better chance to acquire DNA elements, which increase transcriptional activity either by chance or via as-yet uncharacterized biological mechanisms.

To illustrate the evolutionary trajectories of the gene structures and the distribution of the phylogenetic groups, we classified the members of the phylogenetic groups supported by SH-like values of 0.9 or higher (e.g., F1, F2, etc. in Fig. 1) into subgroupings with 0.8 or higher SH-like values (e.g., 0, 1, 2 in Fig. 2A and different colors in Fig. 2B), which formed smaller monophyletic clades in the original monophyletic groups. We then sorted the members of the subgroupings according to the gene models in the genomic context (Fig. 2). As a result, we determined that the degrees to which intergenic or intragenic duplication affected genomic structures were dependent on each genomic locus. Although the LHCF group F1 was the largest grouping recognized in this study and the internal relationships of subgroups were more complicated, the compositions of most genomic loci were explicable by repeated rounds of inter- and intragenic duplications, except for subgroup 0, for which we could not assign phylogenetic affiliations in the tree (Fig. 2B). In addition, the number of *Lhcr*-type gene loci was relatively lower than the *Lhcf*-type, but characteristic fusions of phylogenetically distant paralogs (e.g., fusion of R1, R3 and R4) were conspicuous (Fig. 2A). Some members (i.e., subgroup 1) of LHCR group R3, for example, formed polypeptides with the groups R1 and R4, whereas in another locus, the same type of R3 members (subgroup 3) was tandemly arranged (Fig. 2A). Notably, many of the polyproteins classified in the group F1, but no members of other groups, possess an amino acid sequence motif for the cleavage site between paralogs, called the SPLR motif, which was originally found in some LHC polypeptides in the dinoflagellate *Amphidinium carterae* [8] and *Symbiodinium* sp. C3 [5]. The SPLR motif-containing proteins in these species were phylogenetically associated with the *S*. *minutum* group F1 (data not shown), suggesting that the SPLR motif emerged concomitantly with the diversification of group F1 genes in ancestral dinoflagellates. This pattern also suggests that the cleavage sites of the LHC polyproteins in other phylogenetic groups were divergent in *S*. *minutum*.

**Fig 3:**
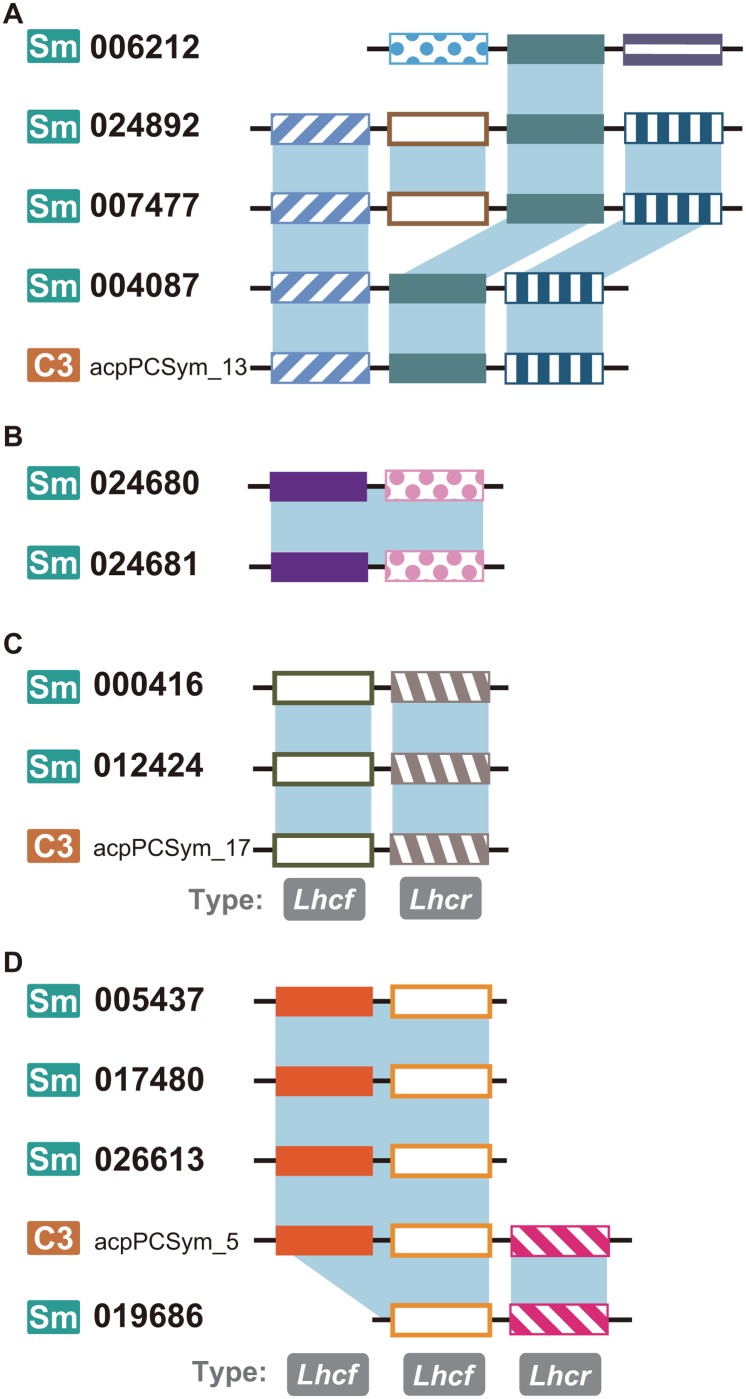
Intra- and intergenic LHC gene duplication in *Symbiodinium*. Bold and non-bold numbers indicate the *S*. *minutum* gene IDs (labeled as ‘Sm’) [20] and the gene names in *Symbiodinium* sp. C3 (‘C3’) [5], respectively. Colored boxes on black bars represent mature protein units in LHC gene loci. Each shaded region indicates the units that are monophyletic within subgroups in Fig. 1.

Fig. 3 shows typical examples of the two types of gene duplications and the comparison with another species, *Symbiodinium* sp. (clade C3). One of the *S*. *minutum* LHC proteins encoded by a gene (Gene ID 006212) in Fig. 3A was also paralogous to one of the protein units in the *S*. *minutum* LHC polyproteins 024892 and 007477. The latter two polyproteins showed partial similarity to another *S*. *minutum* protein, 004087, which was an ortholog of the acpPCSym_13 in *Symbiodinium* sp. C3 [5] (Fig. 3A). Another example in Fig. 3B represents intragenic duplication, in which the gene unit duplicated within the single locus and the multi-unit gene as a whole duplicated next to each other. Those duplicated gene clusters were located at the very end of the scaffold ID 2350 (data not shown) in the *S*. *minutum* genome database [20], and it is possible that there are additional duplicated units in the chromosomal region that the genome sequencing failed to cover. Overall, it seems that these two modes of (inter- and intragenic) gene duplication have both contributed to the unusual expansion of the LHC gene family, especially in the F1 group of the *Lhcf*-type subfamily, which has been extensively duplicated in the *S*. *minutum* nuclear genome (Figs. 1 and 2). These patterns of homology suggest that gene shuffling, including (i) insertion and deletion, (ii) fusion and splitting, and (iii) simple ‘copying and pasting’ of genes in separate loci, may have facilitated the amalgamation of different types of LHC genes into a single polypeptide, resulting in a variety of fine-tuned physiological responses in the diversification of gene repertoire and genome structure.

We also identified fusion genes of *Lhcr*- and *Lhcf*-type LHCs in three gene loci in *S*. *minutum* and two transcripts in *Symbiodinium* sp. C3 (Fig. 3C and 3D). In these cases, considering that both subtypes of LHC genes were separated on the phylogenetic tree (Figs. 1 and 2), it is likely that gene conversion of one subtype into another, or gene fusion between the two, generated the fusion gene of LHCF and LHCR in the common ancestor of the *Symbiodinium* species before the divergence of the genus *Symbiodinium*. One plausible scenario explaining Fig. 3D is that a gene with two *Lhcf*-type and one *Lhcr*-type units (similar to acpPCSym_5) may have been an ancestral form of this group of genes, followed by gene unit loss of either type in the ancestor of *S*. *minutum* and resulting in the current composition of two *Lhcf*-type genes (IDs 005437, 017480, and 026613) and one *Lhcf-* and one *Lhcr*-type gene (ID 019686). Such fusion genes of different types were limited in number and distribution; they were associated with specific phylogenetic clades (F2, R2 and R5), implying that the simultaneous transcription and/or translation of different types of LHC genes may only marginally contribute to the light-harvesting function and perhaps the selective advantage. From an evolutionary perspective, it was previously proposed that gene conversion substantially contributed to the evolution of the LHC genes in *Euglena* [26], and our results suggest that a similar evolutionary mechanism was responsible for the diversified LHC gene family in *Symbiodinium*. This finding represents another example of convergent evolution between euglenophytes and dinoflagellates [9].

We did not detect homologs encoding stress-responsive LHCSR/LHCX families or green plant-type LHCA/LHCB protein families [1,47]. This result is consistent with a hypothesis proposed by Niyogi and Truong, who suggested that a stress-responsive *Lhcsr/Lhcx* family was lost in the common ancestor of extant dinoflagellates [40]. Although another stress-related LHC protein, called PSBS, which contains four trans-membrane helices, has been shown to play a major role in light energy dissipation in the streptophyte green plant lineage [48,49], no PSBS homologs were found in *S*. *minutum*. Moreover, it remains controversial how dinoflagellates cope with high light stress caused by excess light energy absorbed by chlorophylls and other photosynthetic pigments. Reynolds et al. presented a model wherein the dissociation of PCPs from the photosystems attached to LHCs could achieve high light energy dissipation [17], and Kanazawa et al. provided evidence that PCP was not detached from photosystems and, instead, LHC itself functioned as a light energy quencher [18]. It is also important for *Symbiodinium* to acclimate its light-harvesting systems to the high light under elevated temperature, which is proposed to be a physiological factor triggering coral bleaching. Recently, the functional roles of the major carotenoid species in *Symbiodinium* for photoprotection of the reaction center, namely, peridinin and diatoxanthin/diadinoxanthin, were questioned by spectroscopic analysis [6]. In diatoms, which possess fucoxanthin as a major light-harvesting carotenoid instead of the peridinin found in dinoflagellates, it has been shown that multiple *Lhcsr/Lhcx* genes are present in the nuclear genomes and encode LHCX proteins playing a key role in photoprotection [50,51]. Given the presence of *Lhcsr/Lhcx* genes in other algal lineages, such as brown algae and chromerids [40], dinoflagellates may have developed a unique strategy to maintain the photoprotection machinery. Future studies exploring stress responses via LHC expression might explain the absence of *Lhcsr/Lhcx* gene family in dinoflagellates; LHCSR/LHCXs in the ancestral dinoflagellates might have been taken over by independently evolved stress-responsive LHCs and lost during evolution [40].

The photo-induced stress response in *Symbiodinium*, especially on LHC complex maintenance, remains to be investigated to understand how photobleaching occurs and can be prevented in the *Symbiodinium-*cnidarian symbiotic system. In our use of the RNAseq data by Shoguchi et al. [20], which were not originally designed for quantitative analysis but, rather, as a qualitative measure, we did not find drastic changes in the mRNA abundance of genes encoding LHCs (S1 Fig) as well as zeaxanthin epoxidase (ZEP) and violaxanthin de-epoxidase (VDE), which are responsible for the epoxidation and de-epoxidation reactions of diatoxanthin/diadinoxanthin, respectively, in the xanthophyll cycle in many algal species (S2 Fig) [52]. These findings are consistent with previous studies [53–55]. Notably, we found no apparent indications of heat stress-specific responses of other genes encoding proteins that are presumably involved in the heat stress response, namely, heat shock proteins 90 and 70 (Hsp90 and Hsp70), DnaJ-like proteins, and reference genes highly conserved in eukaryotes (S2 Fig). These observations led us to speculate that it may be necessary to examine the heat stress response in different time courses under different physiological conditions and/or that stress response and acclimation processes may be regulated at the posttranscriptional level, which has not been fully investigated and requires attention in the future study of this species and dinoflagellates in general.

**Fig 4:**
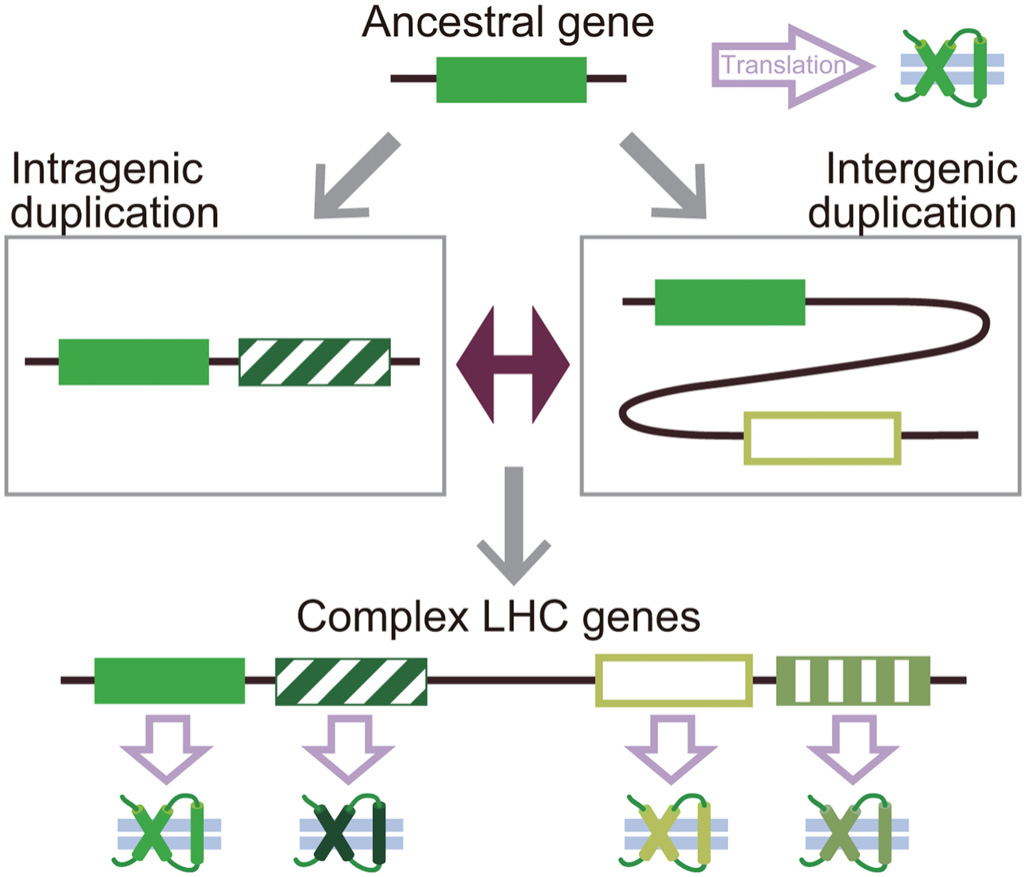
A model evolutionary scenario of LHC gene expansion. In this model, an ancestral LHC gene, which had been translated into a single protein, may have evolved into a multi-unit gene with complex gene structure via multiple rounds of duplication within the gene locus (intragenic, left box) and/or in separate loci (intergenic, right box).

In conclusion, our results provide a well-annotated classification of the LHC genes in *S*. *minutum*, suggesting that the ‘hyper-diversity’ of the LHC gene family has been formed through multiple rounds of intra- and intergenic subunit-based duplication events, most prominently in one of the LHCF subfamilies in this species (Fig. 4). In combination with previous studies [5,20,21], our results highlight the potential for data-mining analysis using whole-genome sequence data to extend our understanding of the diversity of highly redundant multi-gene families such as LHCs [30]. Our results also show that the order and arrangement of LHC proteins are conserved between *S*. *minutum* and *Symbiodinium* sp. C3, suggesting that the basic pattern of gene duplication emerged in the common ancestor of these two strain/species and was established prior to the speciation of *S*. *minutum* or possibly before the divergence of the genus *Symbiodinium* (Fig. 4). In light of the evolutionary trajectory and distribution pattern of the LHCs uncovered by this study, the updated classification of the LHC in *S*. *minutum* will help clarify the assemblies and compositions of the LHC complexes in PSI and II at the protein level in future studies.

## Supporting Information

S1 Fig Maximum likelihood tree of the *S*. *minutum* LHC proteins. An approximate ML tree was generated by FastTree. Thick lines indicate that the branch is supported by both SH-like support values (0.8 or higher) and bootstrap support values (50% or higher) calculated by FastTree and RAxML, respectively, using a matrix containing the LHC proteins from *Symbiodinium minutum* (purple), *Symbiodinium* sp. C3 (blue-green), diatoms (orange) and the green alga *Chlamydomonas reinhardtii* (green). Medium and thin lines indicate that the branch is supported by either or none of those methods, respectively. The RPKM values calculated using the RNAseq data are shown as colored boxes. Asterisks indicate the genomic loci, showing very low RPKM values except for the ‘Control 72 hour’ samples.
(PDF)

S2 Fig Relative mRNA abundance of conserved nuclear-encoded genes is not drastically altered under heat stress. Homologs of genes encoding conserved proteins, including zeaxanthin epoxidase (ZEP), violaxanthin de-epoxidase (VDE), heat shock proteins (HSP) 90 and 70, DnaJ-like protein, actin, ß-tubulin (TubB) and elongation factor-like protein (EF-like), were used to calculate the relative abundance of mRNA accumulation based on the RPKM values from the RNAseq data.
(PDF)
