# Supplementary material for: Diversification of the Light-Harvesting Complex Gene Family via Intra- and Intergenic Duplications in the Coral Symbiotic Alga Symbiodinium
Source: PLoS One. 2015 Mar 5;10(3):e0119406. doi: 10.1371/journal.pone.0119406 (PMC4351107; doi:10.1371/journal.pone.0119406)
Supplement: S1 Fig — An approximate ML tree was generated by FastTree. Thick lines indicate that the branch is supported by both SH-like support values (0.8 or higher) and bootstrap support values (50% or higher) calculated by FastTree and RAxML, respectively, using a matrix containing the LHC proteins from Symbiodinium minutum (purple), Symbiodinium sp. C3 (blue-green), diatoms (orange) and the green alga Chlamydomonas reinhardtii (green). Medium and thin lines indicate that the branch is supported by either or none of those methods, respectively. The RPKM values calculated using the RNAseq data are shown as colored boxes. Asterisks indicate the genomic loci, showing very low RPKM values except for the ‘Control 72 hour’ samples. (PDF) [file pone.0119406.s001.pdf]

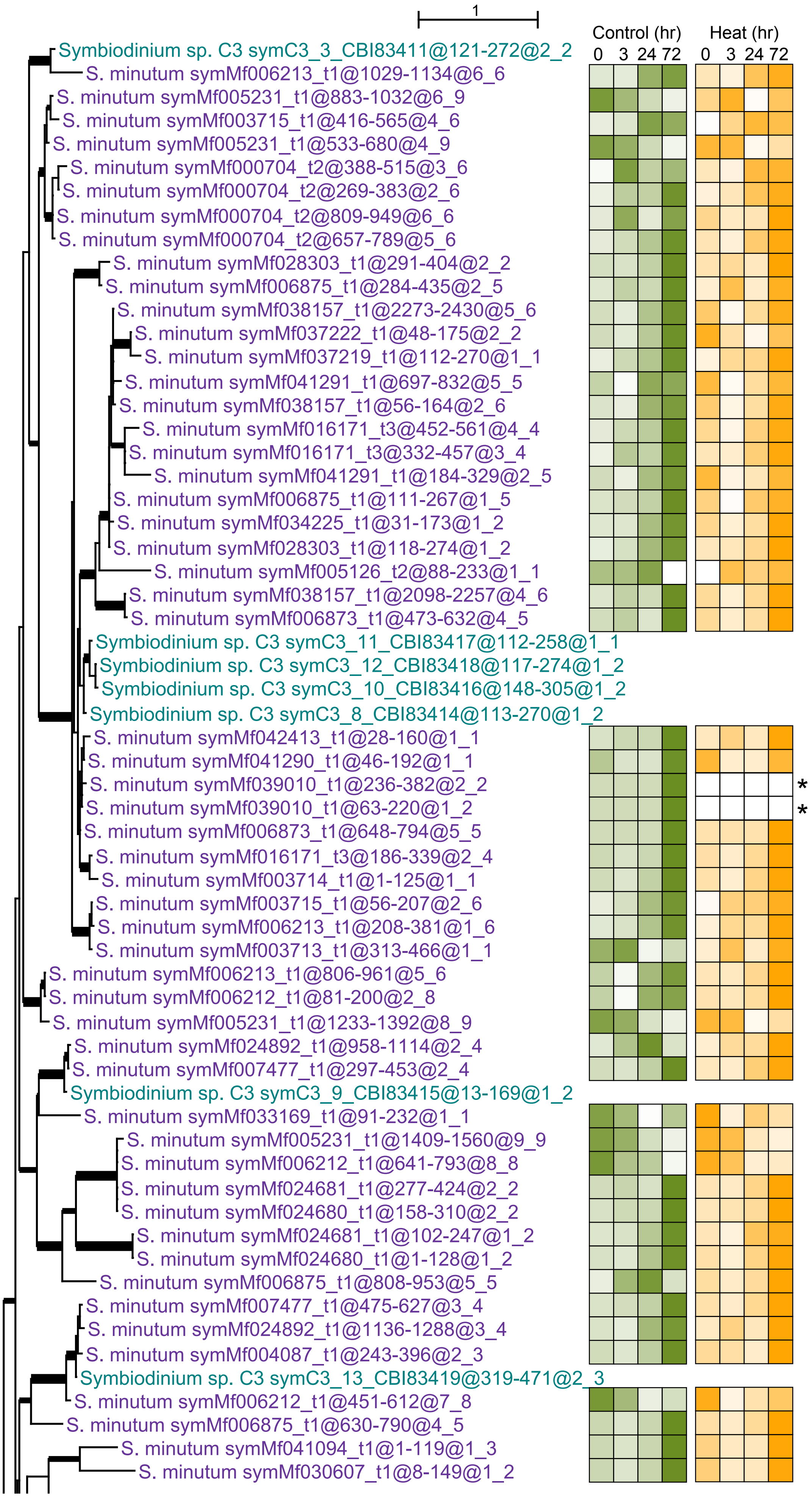

Maruyama et al. Figure S1 (Continued)

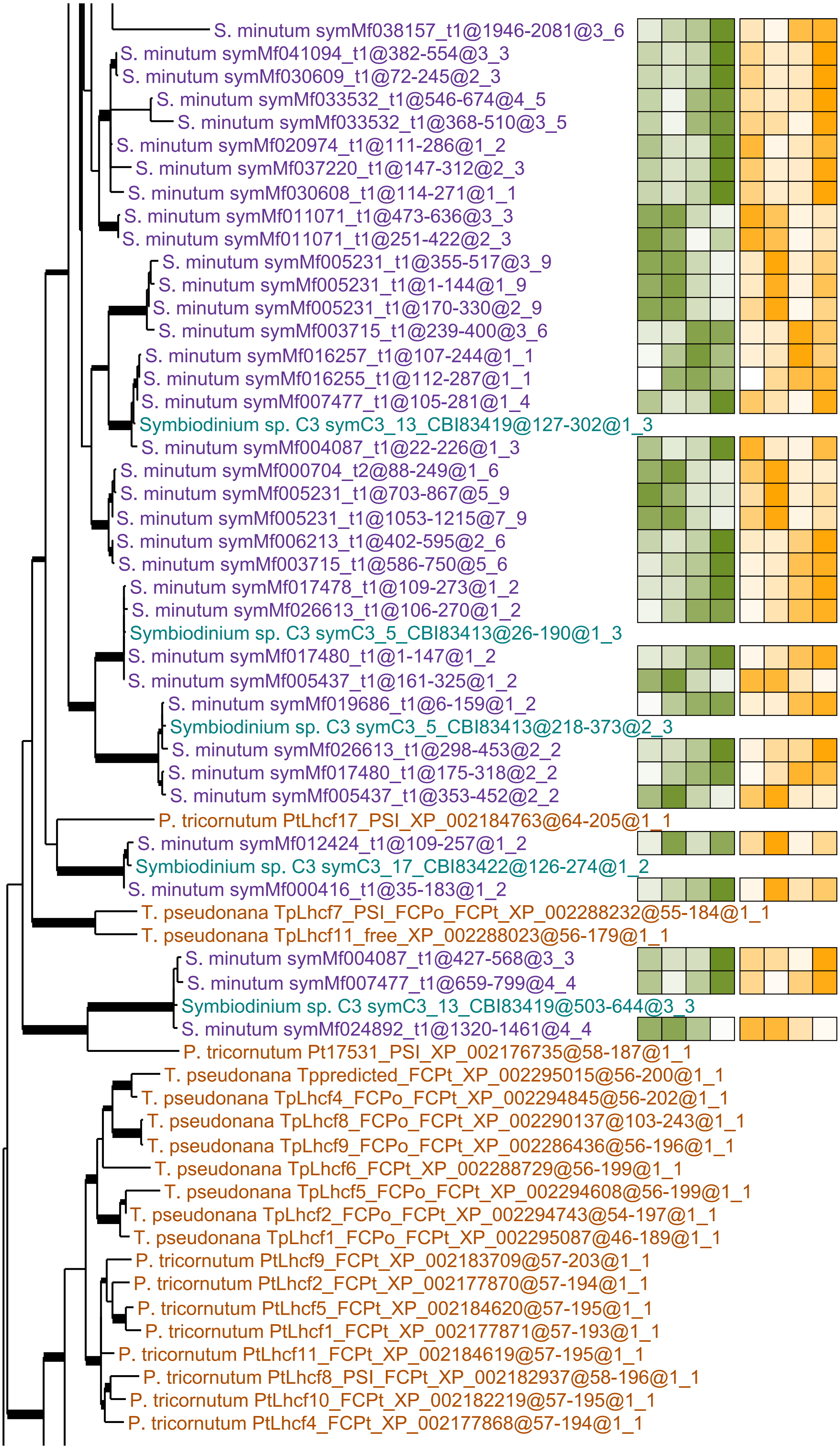

Maruyama et al. Figure S1 (Continued)

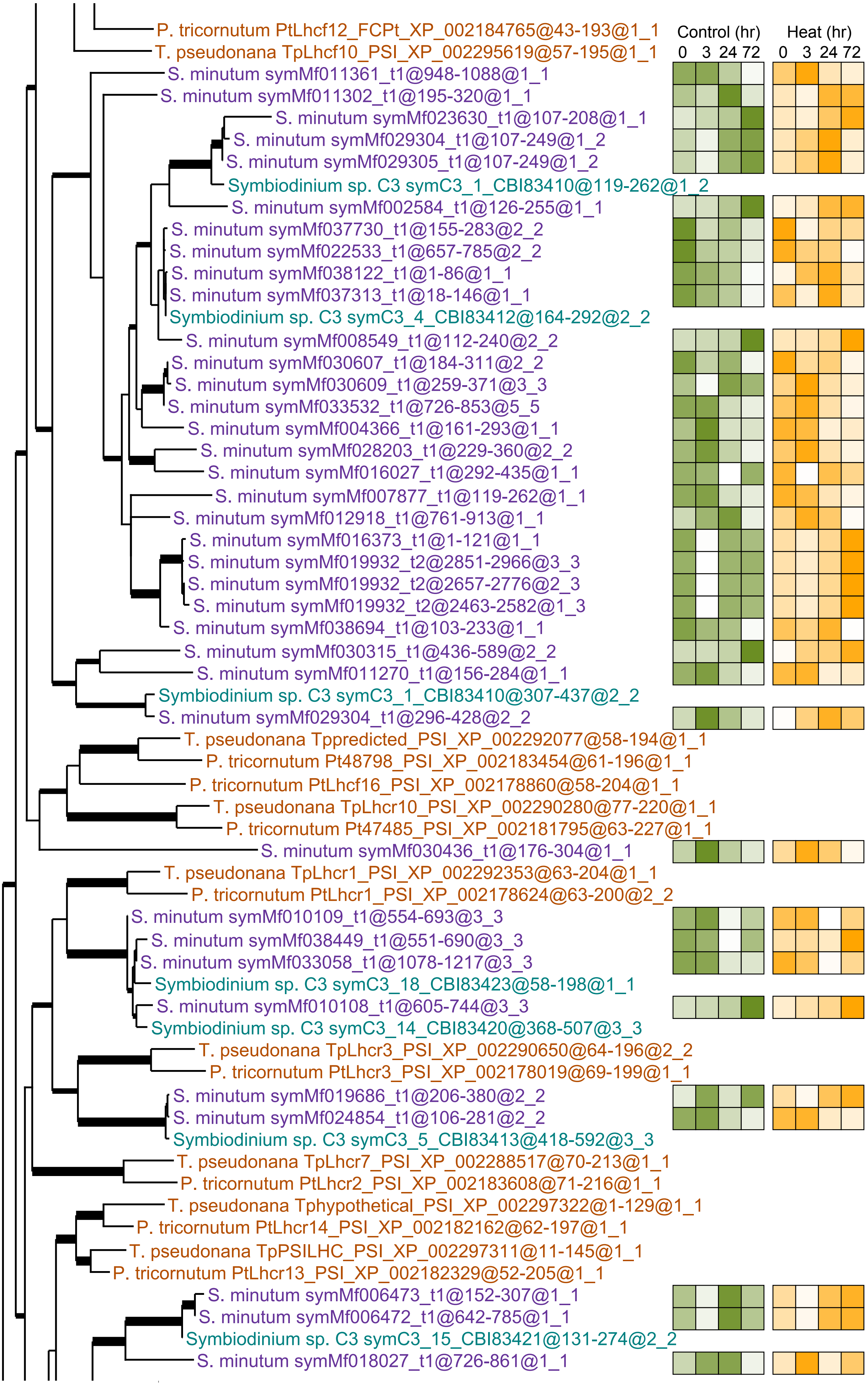

Maruyama et al. Figure S1 (Continued)

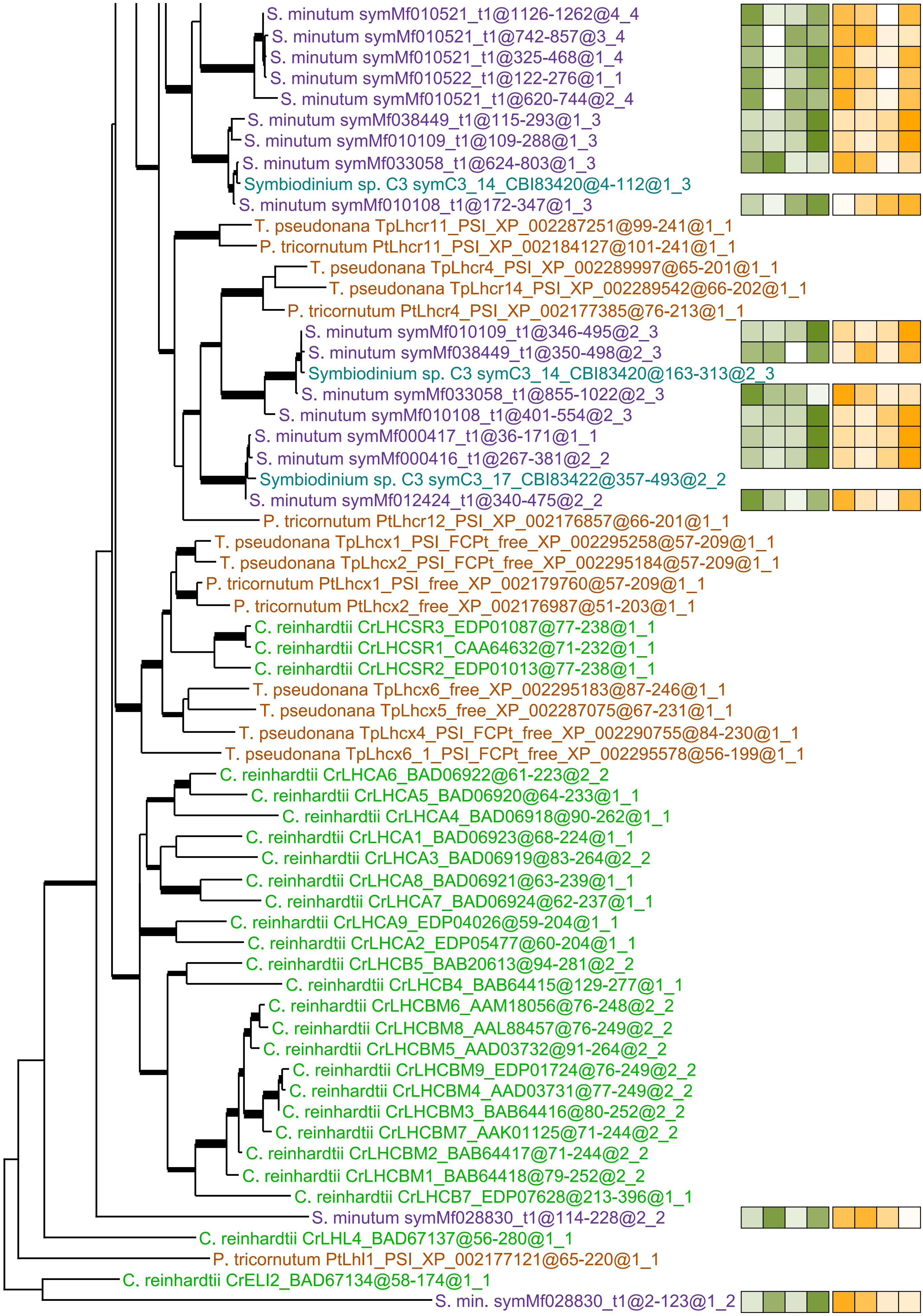

Maruyama et al. Figure S1
