# Supplementary figures and images for: Diversification of the Light-Harvesting Complex Gene Family via Intra- and Intergenic Duplications in the Coral Symbiotic Alga Symbiodinium
Source: PLoS One. 2015 Mar 5;10(3):e0119406. doi: 10.1371/journal.pone.0119406 (PMC4351107; doi:10.1371/journal.pone.0119406)

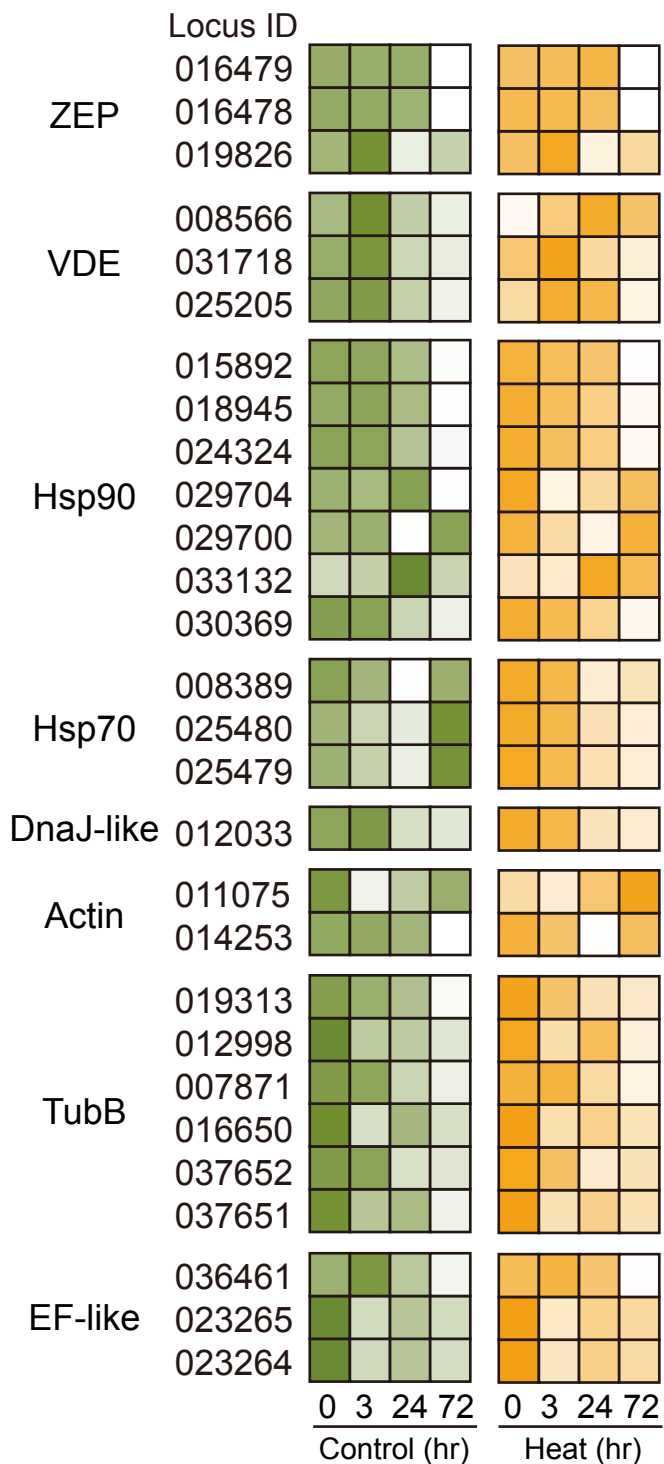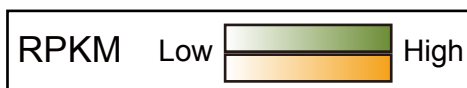

Supplement: S2 Fig — Homologs of genes encoding conserved proteins, including zeaxanthin epoxidase (ZEP), violaxanthin de-epoxidase (VDE), heat shock proteins (HSP) 90 and 70, DnaJ-like protein, actin, ß-tubulin (TubB) and elongation factor-like protein (EF-like), were used to calculate the relative abundance of mRNA accumulation based on the RPKM values from the RNAseq data. (PDF) [file pone.0119406.s002.pdf]
